# Cryptococcal meningitis in a goat – a case report

**DOI:** 10.1186/1746-6148-10-84

**Published:** 2014-04-05

**Authors:** George Stilwell, Hugo Pissarra

**Affiliations:** 1 Centro de Investigação Interdisciplinar em Sanidade Animal, Faculdade de Medicina Veterinária, University of Lisbon, Alto da Ajuda, Lisbon 1300-477, Portugal

**Keywords:** Cryptococcus, Meningitis, Goat, Allodynia, Ataxia, Caseous lymphadenitis

## Abstract

**Background:**

*Cryptococcus spp.* are saprophytic and opportunistic fungal pathogens that are known to cause severe disease in immunocompromised animals. In goats there are reports of clinical cryptococcal pneumonia and mastitis but not of meningitis.

**Case presentation:**

The following report describes a case of a five year old buck showing severe neurological signs, including paraplegia and strong pain reaction to touch of the hindquarters region. Treatment with antibiotics was unsuccessful and the animal was euthanized for humanitarian reasons. Postmortem examination revealed lumbar meningitis, lung nodules and caseous lymphadenitis lesions. Encapsulated *Cryptococcus neoformans* were identified from the lungs and meninges, showing that cryptococcal meningitis should be included in the differential diagnosis of goats showing paresis and hyperesthesia. The possibility of concurrent immunosuppression due to *Corynebacterium pseudotuberculosis* infection is raised.

**Conclusions:**

Cryptoccocal meningitis should be included in the differential diagnosis list of goat diseases with ataxia and hyperesthesia.

## Background

*Cryptococcus sp*. is a dimorphic yeastlike fungus with worldwide distribution. In animal tissues Cryptococcus is seen as a round encapsulated organism sometimes forming daughter buds that are connected to the parent cell by an isthmus. The capsule provides protection from the host immune response and favours chronic infection and dormancy [1]. *Cryptococcus neoformans* is the usual causative agent of animal cryptococcosis. *C. gattii*, restricted to tropical and subtropical regions, was previously classified as a variety of *C. neoformans* but is now considered an independent species. *C. neoformans* causes disease in both animals and humans and is usually linked to immunosuppressive diseases in humans (HIV infection) and cats (FeLV and FIV), commonly presenting signs as the immunosuppressed patient’s immunity wanes. The organism has been frequently isolated from pigeon guano where it may remain viable for at least two years. *C. gatti* has been found to be especially associated with decomposing material from eucalyptus trees [2,3].

There is strong evidence that the mode of infection is via inhalation of air-borne organisms (desiccated yeast cells or spores) and that systemic cryptococcosis will start in the nasal cavity or the lungs, although respiratory involvement usually does not result in clinical signs. In the immunocompetent host, infection is usually limited to the lungs and is asymptomatic [4]. *C. neoformans* has predilection for the central nervous system with spread being probably haematogenous or by sinonasal extension to the CNS through the cribriform plate [5,6]. Skin disease in humans, cats and dogs [3] and sinonasal and ear infection in cats have been described [7]. In ruminants the organisms may also enter through the teat causing mastitis.

Our report describes a case of a male goat with neurologic signs that was diagnosed post-mortem as having fungal (Cryptococcus) meningitis.

This study was approved by the Welfare and Ethics Committee of the Interdisciplinary Centre of Research in Animal Health of the Lisbon Veterinary Faculty (CIISA-FMV).

## Case presentation

A 5 year old male goat (from an endangered Portuguese breed called Bravia) was presented with depression, reduced appetite and hind limb incoordination. Trauma had been suspected and the animal had been treated with an anti-inflammatory drug (carprofen, 2 mg/kg SC) for three days. This pure bred buck was part of a herd of autochthonous animals used to create a national gene bank. Pigeons had access to both the stable and the pastures used by the herd but no other animal had previously shown any neurological or respiratory signs.

**Figure 1:**
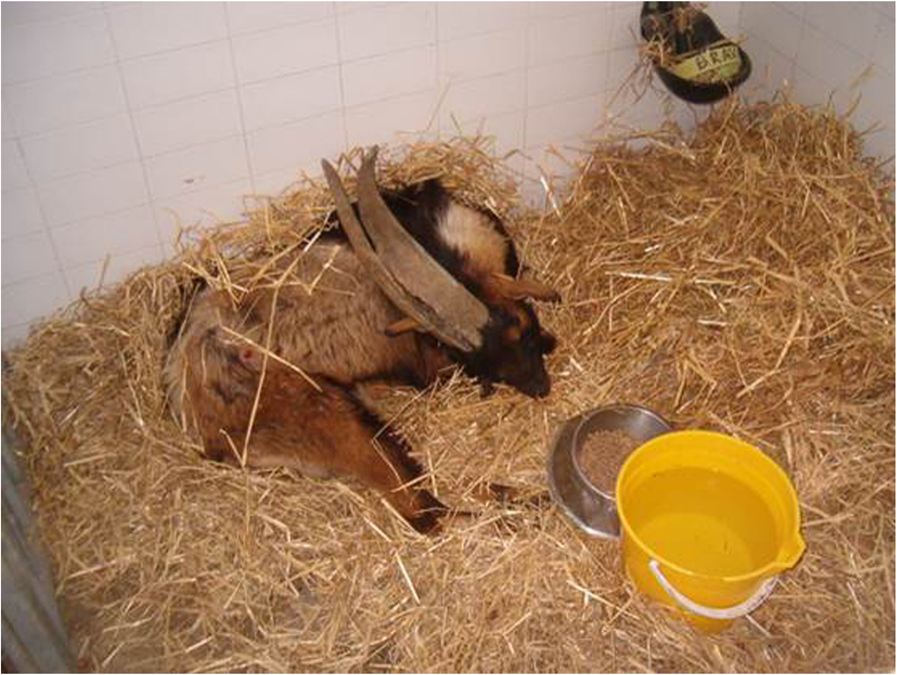
Neurologic signs - buck showing signs of hind limb paralysis and self mutilation (skin excoriation on abdominal flank) due to hyperaesthesia.

The neurological signs were confined to the hindquarters and ataxia rapidly progressed to a complete flaccid paraplegia (Figure 1). Patellar, perineal and hind limb withdrawal reflexes were absent. Severe hyperalgesia and an intense pain reaction to touch were evident on both flanks and the hindquarters – for example, gentle stroking of the hind legs caused high-pitched bellowing. Constant scratching and self-mutilation occurred with the long horns resulting in a large alopecia on the right abdominal wall. There were no seizures, signs of blindness or other cranial nerve deficits. Head movements, reaction to touch and front legs’ muscle tone were reduced but considered normal due to the depression state of the animal, so the neurological examination suggested a lesion limited to the lumbosacral region.

Rectal temperature, heart and respiratory rates were inside the reference ranges. Blood analysis revealed leucocytosis characterized by a neutrophilia and monocytosis, moderate anemia and hyperproteinemia (Table 1). Renal and liver values were normal. Radiography of the lumbosacral region were unremarkable. Cerebrospinal fluid (CSF) was collected by lumbar puncture under local anaesthesia. The fluid was clear, colourless and did not flow freely from the needle hub. CSF analysis showed severe mixed pleocytosis (980 cells/mm^3^; 65% neutrophils and 35% lymphocytes) with mild increase in protein concentration (1.15 g/L). No cryptococcal organism was identified and bacteria culture of the CSF yielded no growth. Meanwhile treatment was initiated with cephalosporin (ceftiofur, 4 mg/kg IM twice daily, Pfizer) and dexamethasone (one single 0.25 mg/kg IM injection). After three days of antibiotic treatment (six days after initial clinical signs) hyperesthesia continued to be very severe and so the buck was euthanized for humanitarian reasons.

**Table 1:** Blood parameters of male goat with neurological signs secondary to meningitis

**Parameters**	**Buck values**	**Reference range**^**a**^
WBC (×10^3^)/μl	17.2	4-13
Erythrocytes (×10^6^)/μl	6.71	8-18
Hgb (g/dL)	6.97	8-12
PCV (%)	13	22-38
MCV (fl)	19.3	16-25
MCH (pg)	10.4	5.2-8
MCHC (g/dL)	53.8	30-36
Neutrophils (mature)	13,072 (76%)	12,000-7,200 (30-48%)
Lymphocytes	3,268 (19%)	2,000-9,000 (50-70%)
Monocytes	860 (5%)	0-550 (0-4%)
Total Proteins (g/dl)	8.1	5-7.5

^a^Merck Veterinary Manual.

**Figure 2:**
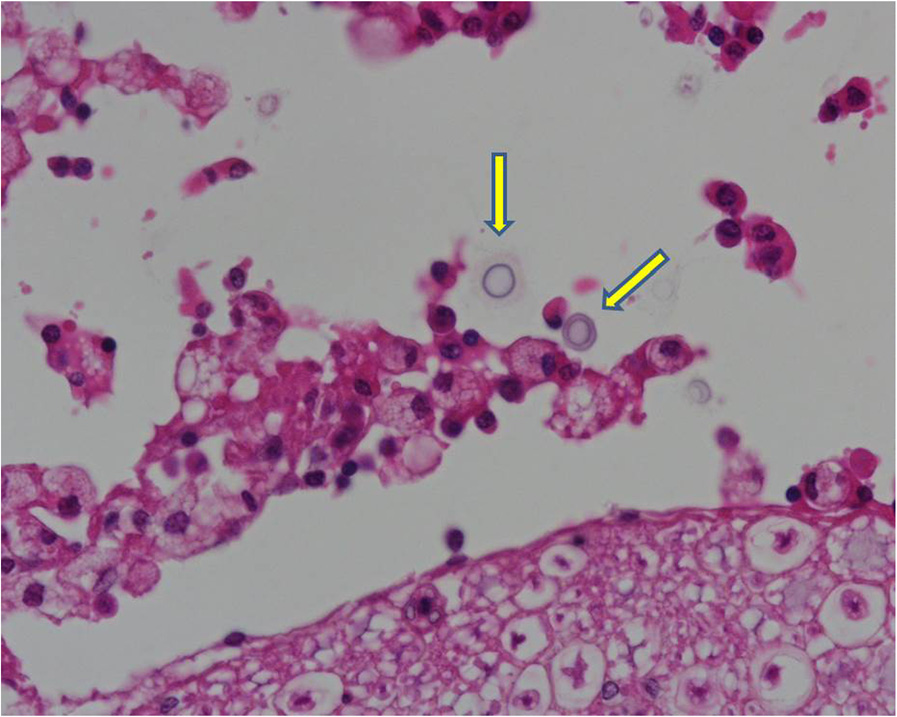
**Histopathology exam - several round encapsulated microorganisms (yellow arrow) isolated from the lumbar meninges were identified as being *Cryptococcus neoformans *** **(HE, ×400).**

Post-mortem examination revealed cachexia, hind limbs muscular atrophy and skin excoriations on both flanks. Mild pneumonia in the ventral lobes of the right lung, two well demarcated nodules with 1 cm in diameter in the right lung, caseous material in mediastinum and peripheral lymph nodes and an enlarged oedematous lumbar spinal cord were evident on gross internal examination. Microscopically there were signs of granulomatous pneumonia and extensive congestion and oedema with lymphocyte infiltration of the lumbosacral meninges consistent with acute meningitis. In both lung and meninges numerous round shape organisms with narrow-necked budding were visible and identified morphologically as *C. neoformans* (Figure 2). *Corynebacterium pseudotuberculosis* was isolated from the caseous material found in the lymph nodes.

## Discussion

Pulmonary disease and mastitis have been described in sheep and goats, but no report of goat meningitis due to *Cryptococcus* infection has been published. Published references to caprine cryptococal disease include an outbreak of severe pneumonia by *C. gattii* in Spain, cryptococcal pneumonia associated to *M. bovis* infection, experimental and natural occurring clinical mastitis by *C. neoformans* and a granuloma obstructing the nasal cavity of a goat in Australia [8-12].

Non-specific clinical findings may lead to mis-diagnosis because other infectious diseases are much more common in small ruminants. The differential diagnosis in our case of lumbosacral meningitis should include other neurological diseases causing lower spinal cord lesion leading to hindquarters paresis and hyperestesia [13]. Listeriosis and polioencephalomalacia may cause ataxia, recumbency and depression but will usually present cranial nerve deficits and central blindness. Scrapie is more common in sheep but it does occur in goats causing ataxia and intense pruritus. Other less likely causes of ataxia, paraplegia or central hyperestesia are bacterial meningitis (*E. coli*, *Pasteurella sp., Mycoplasma sp.*), inter-vertebral disc hernia and trauma (e.g. vertebral fracture), rabies, tetanus or meningitis secondary to extension of superficial lymph node infection by *Corynebacterium pseudotuberculosis* or *Arcanobacterium pyogenes*.

A definitive diagnosis requires isolation of *C. neoformans* from blood or body fluids such as CSF. Cryptococcal antigen latex agglutination serology (CALAS) can be performed on serum or body fluids but only provides presumptive evidence [14]. However, in our case isolation from the CSF was not possible suggesting that negative results should not preclude the hypothesis of a cryptococcal infection of the meninges. Our results also suggest that an ante-mortem diagnosis may be difficult and so prompt anti-fungal treatment should be implemented if no response to antibiotics is evident.

Prompt treatment with amphotericin B plus flucytosine, fluconazole, itraconazole or ketoconazole, have shown some success in the treatment of cats, horses and humans. Success with oral fluconazole (5 mg/Kg/day PO for 6 months) was established in a goat with abdominal wall infection with *C. gatti*[15]. However, the prognosis for infected animals that develop CNS signs is considerably worse and humane euthanasia may be warranted.

Although cryptococcosis is considered to be typically a chronic infection, in our case severe unusual neurological signs developed within days. It was not possible to estimate the duration of the lung infection but it should stressed that no respiratory signs were evident at clinical examination of this animal. Strains differ in their virulence, but the immune status of the host seems to be more important than the virulence of the strain [2]. Systemic disease in other animals have been associated with immunosuppressing factors, such as HIV, Feline immunodeficiency virus (FIV), Feline leukemia virus (FeLV) and surgery [2]. Gutiérrez and García Marin suggest an underlying immunodeficiency in a goat with granulomatous pneumonia caused by *C. neoformans* together with *M. bovis*, that was negative on intradermal and serologic tests for tuberculosis [9]. There are reports of synergism between *C. pseudotuberculosis* and other bacteria or virus (e.g. Maedi-visna), predisposing animals to severe pneumonia and other diseases [16]. Suggesting involvement of the caseous lymphadenitis in the pathogenesis of this cryptococcal meningitis is speculative but deserves mentioning.

## Conclusions

While *Cryptococcus spp.* has been previously reported to cause mastitis and pneumonia in goats, central nervous system infection has not been demonstrated in this species. The clinical relevance is that cryptoccocal meningitis should be included in the differential diagnosis list of goat diseases with ataxia and hyperesthesia.

## Competing interests

The authors declare that they have no competing interests.

## Authors’ contributions

GS followed the clinical case and drafted the manuscript. HP carried out the necropsy and did the pathology report. Both authors read and approved the final manuscript.
